# Appraisal of the water footprint of irrigated agriculture in a semi-arid area: The Segura River Basin

**DOI:** 10.1371/journal.pone.0206852

**Published:** 2018-11-06

**Authors:** José Miguel Martínez-Paz, Francisco Gomariz-Castillo, Francisco Pellicer-Martínez

**Affiliations:** 1 Department of Applied Economics, University of Murcia, Murcia, Spain; 2 Department of Geography, University of Murcia, Murcia, Spain; 3 Department of Civil Engineering, Catholic University of Murcia, Murcia, Spain; Leiden University, NETHERLANDS

## Abstract

Irrigated agriculture is a key activity in water resources management at the river basin level in arid and semi-arid areas, since this sector consumes the largest part of the water resources overall. The current study proposes a methodology to evaluate the water footprint (WF) of the irrigated agriculture sector at the river basin level, through a simulation of the anthropised water cycle combining a hydrological model and a decision support system. The main difference from the approaches that have already been used is that the new methodology includes the limitations of the system for the exploitation of water resources where the irrigated areas are located, and it considers the hydrological principles governed by the law of continuity of mass. Water footprint accounting was carried out for the Segura River Basin (South-eastern Spain), applying the methodology proposed and another that is usually applied. The results of the two methodologies were compared, revealing significant differences in the values of the WF, basically due to the blue component. The methodology that is usually applied overestimated the WF of the agriculture in the basin since supply deficits were not taken into account, providing results that would only be possible if there were no spatial or temporal restrictions to water use. So, in order to make the WF indicator useful in water resources management plans, it is necessary to adapt the computations to the main characteristics of the water exploitation system of the whole basin under study, respecting the hydrological principles of the water cycle: regulation and transport infrastructure, the real water resources available and the priority of access to water between concurrent water uses.

## Introduction

The scarcity of and/or pollution in continental fresh water is currently one of the main issues regarding natural water resources management at the global level [1], particularly in semi-arid areas with low availability of resources and concurrent water uses [2]. The assessment of these available water resources and their use is a priority for the authorities in charge of water allocation, which is generally developed at the basin level [3]. Agriculture is, by far, the sector that demands the largest water volume at the global level, accounting for no less than 70% of the water used in the world [4]. Therefore, the analysis of water use in agriculture is imperative for water resources management policies.

The water footprint (WF) is an indicator that measures the use of fresh water as a production factor at different levels (goods, services, businesses, geographical areas and so on). It takes into consideration water sources, giving rise to three components represented by colours: green, blue and grey [5]. The green component is the consumption of the precipitation that is stored in the root zone of the soil by plants (WF_Green_). The majority of this consumed water is lost from the plants by evapotranspiration, although a small part can be incorporated into the plants. The blue component is water that has been sourced from surface or groundwater resources and either evaporates, is incorporated into a product or taken from one water body and returned to another or is returned to the same basin but in a time period different from that of the analysis being carried out (WF_Blue_). The grey component is the amount of fresh water that would be required to assimilate any pollution and meet specific quality standards (WF_Grey_) [6]; this volume does not represent actual consumption. Thus, the WF quantifies the pressure of human activity on water resources, providing results in terms of fresh water volume (homogeneous unit), and can be employed as an indicator of the sustainability of the spatial-temporal management of this resource at the local or regional scale [7]. Therefore, the WF is a multi-dimensional indicator that is particularly adequate for the comprehensive assessment of water use in agricultural activities [8].

In fact, due to the important role of irrigation in the use of water resources and the potential of the WF indicator, a large number of practical applications of WF accounting have been developed [9, 10]. Studies generally use a soil water balance model, in which the value of crop evapotranspiration due to precipitation is the WF_Green_, whereas the irrigation water consumed by crops (evapotranspiration) is the WF_Blue_ [11, 12]. In some cases, the water applied is considered instead of the consumption by the plant, and the leaks and leachates which are not consumed by crops have been called the white water footprint [13]. Finally, the WF_Grey_, which has not been considered in most studies [14, 15] until recently, is generated by the agrochemicals (fertilisers, pesticides, etc.) that reach and hence pollute surface and groundwater [16].

Despite the large number of studies on the WF in agriculture, there are criticisms of the methodologies applied and its usefulness in the management of water resources [17–19]. These derive from the limitations of the methodologies that normally are applied to determine this indicator (see [14, 20], among others). For local or regional cases, the following stand out: it is assumed that there are always blue water resources for an optimal supply and that these resources always have hydraulic infrastructure that enables them to reach the irrigated areas. So, the blue and green components are evaluated separately, ignoring the hydrological principles of continuity of mass. If these limitations are ruled out in a scenario with competition for the same blue water resources, this results in an overestimated value of WF_Blue_, even more so when considering that irrigation has a lower priority than the urban water supply [19], as is usually the case in resource allocation policies [21].

So, all this can lead to a WF value that does not represent reality and cannot be used as a water resources management indicator. In this sense, the main purpose of the current work is to develop a methodology for the accounting of the WF of irrigated agriculture in a specific geographical area that deals with the above-mentioned limitations. This WF accounting is structured on the results from the simulation of the anthropised water cycle of a river basin [22], which links modelling of the natural hydrology to the exploitation system of water resources (hydraulic infrastructure, available water resources, supply sources, priority between water uses, etc.). Therefore, although this WF accounting takes into consideration the influence of the soil on plants, it is done from the engineering perspective of water resources management. In addition, the results obtained with the proposed methodology are contrasted with those obtained with one of the methodologies that has been applied elsewhere, in which there are no water deficits for irrigation.

Both methodologies were applied to the Segura River Basin (SRB), one of the most complex territorial units of water resources management in Europe [23]. This Mediterranean coastal area has a semi-arid climate, the annual average potential evapotranspiration being more than twice the average annual precipitation [24]. In addition, in this region there is intensive use of water, not only for agricultural irrigation purposes, but also for urban supply, tourism and industry, which demand ever larger volumes for their development [25]. The low availability of natural water resources in the SRB has led to a complex supply system and, most importantly, to the assignation of smaller volumes of water to some areas of irrigated crops that would receive greater volumes under optimal supply conditions [26]. Therefore, this case study, beyond its regional interest, is a clear example of irrigated agriculture located in a semi-arid area that is very productive in general terms but has water availability as one of its limiting factors [27].

## Materials and methods

### Crop water footprint accounting

The accounting of the water footprint of irrigated agriculture (WFIA) in a geographical area is based on the estimation of the WF of the crops existing in it for a given period [6]. First, the WF of the main processes that consume or pollute water is estimated for each crop (Eq 1). These processes are mainly the evapotranspiration of rainwater and irrigation water, the accumulation of water in plants and their products (which is not usually taken into account in the final calculation due to its relatively low importance) and the use of agrochemicals. Depending on the aim and scope of the study, and the baseline information, there are different approaches to WF accounting in the specialized literature, from the application of monthly modelling for large areas [28–30] to the study of small plots through daily monitoring [31]. However, in all of them, rainwater evapotranspiration is the green component of the WF (WF_Green_ [volume/time]), the net irrigation water consumed or evapotranspired is the blue component (WF_Blue_ [volume/time]) and the excess of fertilisers/pesticides that ends up in water bodies determines the grey part (WF_Grey_ [volume/time]). Once the WF has been assessed for each crop, the results are aggregated for all of the area under analysis [8, 14, 20].

$$\text{WF}={\text{WF}}_{\text{Green}}+{\text{WF}}_{\text{Blue}}+{\text{WF}}_{\text{Grey}}\left[\text{volume}/\text{time}\right]$$ (1)

The green and blue components are usually evaluated using soil water balance models, such as CROPWAT, based on the approach of [32], AquaCrop [33] or CropSyst [34]. In these models, crop evapotranspiration under standard conditions (ET_c_) is calculated from climatic variables (ET_0_) and agronomic data (K_c_). If ET_c_ is greater than the effective precipitation, then the effective precipitation is the green component of the water consumed (CWC_Green_) and coincides with the evapotranspiration under non-standard conditions (ET_c,adj_). But, if ET_c_ is lower than the effective precipitation, then the CWC_Green_ is ET_c_ (Eq 2). When ETc—ET_c,adj_ is higher than 0, there is a water deficit for the crops. This water deficit (also called the crop water requirement) can be compensated by irrigation and is the blue component of the water consumed (CWC_Blue_) (Eq 3) [6]. Water deficits can be totally covered if one assumes optimal conditions for each crop [14], and the sum of the green and blue components is equal to ET_c_. But, there is also a more realistic option when establishing an irrigation programme for each crop [12], and the sum of the green and blue components may be less than ET_c_ [17].

$${\text{WF}}_{\text{Green}}={\text{CWC}}_{\text{Green}}=\text{min}\left({\text{ET}}_{\text{c}},{\text{ET}}_{\text{c},\text{adj}}\right)\left[\text{volume}/\text{time}\right]$$ (2)

$${\text{WF}}_{\text{Blue}}={\text{CWC}}_{\text{Blue}}=\text{max}\left(0,{\text{ET}}_{\text{c}}-{\text{ET}}_{\text{c},\text{adj}}\right)\left[\text{volume}/\text{time}\right]$$ (3)

The grey component of the WF (WF_Grey_) is calculated by dividing the pollutant load (L [mass/time]) of each *k* pollutant by the difference between the ambient water quality standard for that pollutant (c_max_ [mass/volume]) and its natural concentration in the receiving water body (c_nat_ [mass/volume]). The variable c_max_ is the maximum or limit concentration of each *k* pollutant that the receiving water body is able to assimilate, and c_nat_ is the concentration of each *k* pollutant that would occur if there were no human disturbances in the catchment where the water body is located (Eq 4). The equation is evaluated for every pollutant, and the one that requires the greatest volume for its assimilation (max[k]), known as the critical pollutant, determines the value of WF_Grey_ [6, 35].

$${\text{WF}}_{\text{Grey}}=\text{max}\left[\text{k}\right]\frac{\text{L}\left[\text{k}\right]}{\left({\text{c}}_{\text{max}}\left[\text{k}\right]-{\text{c}}_{\text{nat}}\left[\text{k}\right]\right)}\left[\text{volume}/\text{time}\right]$$ (4)

### Water footprint of irrigated agriculture with full supply (WFIA-FS)

The methodology defined, assuming optimal conditions for crops, and applied in this study is referred to as WFIA-FS (Water Footprint of Irrigated Agriculture with Full Supply) [14]. This methodology is usually applied for the crop pattern of a given year, and the soil balance is simulated using the climatic data for that year. In this case, the crop pattern of a given year was fixed and simulated for the climatic data of a period (several consecutive years). Thus, the climatic variability, which is a determining factor in the calculation of the WF in agriculture, is included in the results.

So, the soil water balance used to calculate the WF was defined with the distributed hydrological model SIMPA [36]. This model simulates, monthly, the water cycle along a period of time. The input data include climate series of precipitation (P) and the crop evapotranspiration under standard conditions (ET_c_), calculated from the reference crop evapotranspiration (ET_0_) and crop coefficients (K_c_). The results are ET_c,adj_ series distributed in cells. So, the CWC_Green_ and CWC_Blue_ (water deficit) series are calculated from the ET_c_ and ET_c,adj_ series of the cells where crops are located (Eqs 2 and 3).

The grey component of the WF (WF_Grey_) is calculated by the following expression (Eq 5):

$${\text{WF}}_{\text{Grey}}=\text{max}\left[\text{k}\right]\frac{\left(\alpha \left[\text{k}\right]\times \text{AR}\left[\text{k}\right]\right)}{\left({\text{c}}_{\text{max}}\left[\text{k}\right]-{\text{c}}_{\text{nat}}\left[\text{k}\right]\right)}\left[\text{volume}/\text{time}\right]$$ (5)

**Fig 1:**
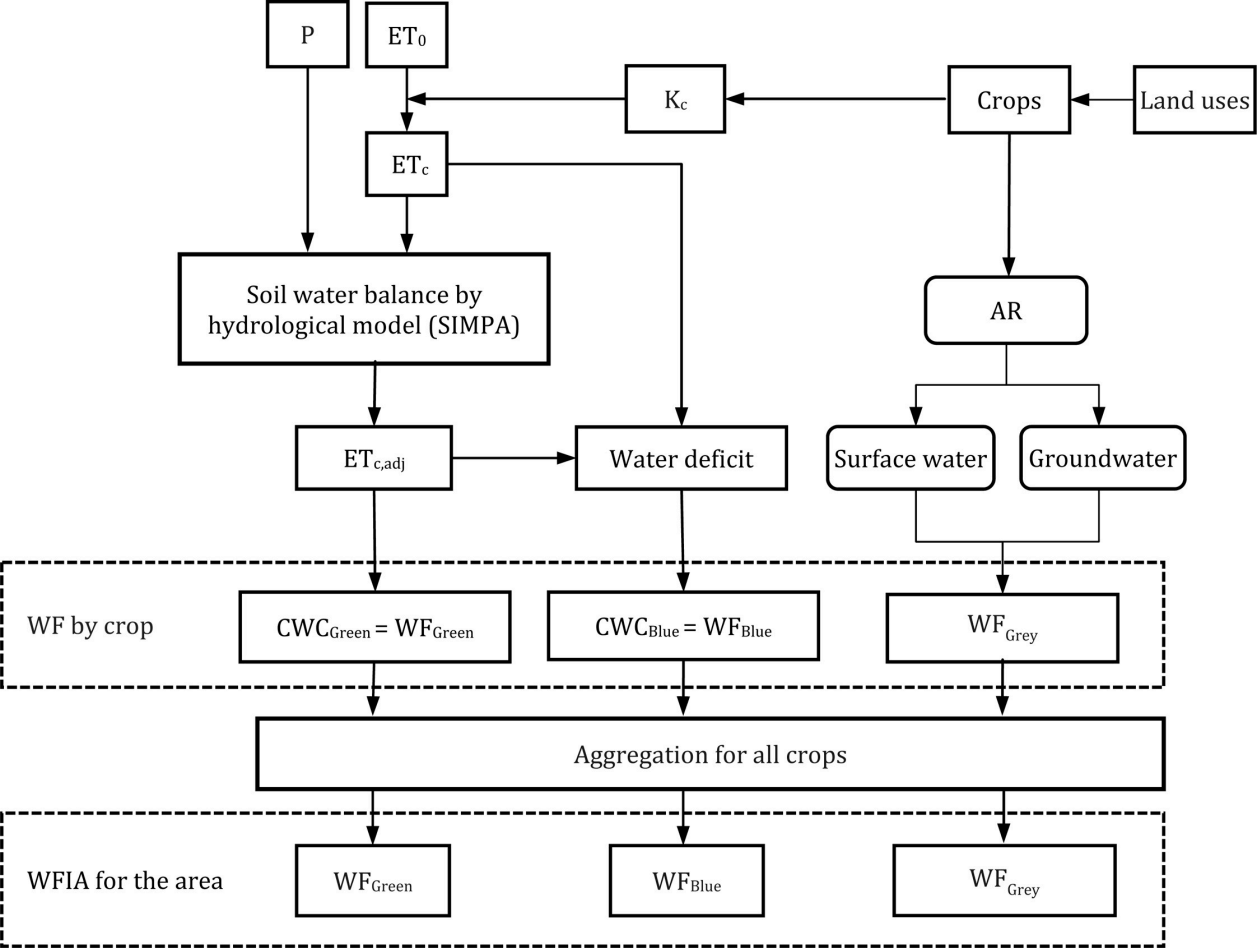
Methodological scheme for WFIA-FS accounting.

This equation assumes that the pollutant load (L) reaching a water body is a percentage (α [%]) of the quantity of agrochemicals (AR [mass/time]) applied to the crops [16], as fertilisers/pesticides. For this case, the k pollutant loads considered are nitrate and phosphate due to fertilisers, taking into account the monthly pattern of their application. Although there are other pollutants in the return flows [37], these are the main source of contamination caused by irrigation in the water bodies [12]. This α percentage depends on the climate, soil, agricultural practices, slope and runoff [16, 34]. In this work, the variables considered to calculate this percentage were the slope of each cell and the amount of irrigation water applied in each cell every month [8, 29, 38]. The volume of water applied, instead of runoff, is used since it is irrigated agriculture. As for the water bodies that receive the pollutants, two possibilities have been considered: irrigated crops located in river plains that discharge directly into the river (surface water bodies), or discharges of crops located far from a natural streamflow that end up polluting aquifers (groundwater bodies) (Fig 1).

### Water footprint of irrigated agriculture with exploitation system (WFIA-ES)

The methodology proposed in this study is called Water Footprint of Irrigated Agriculture with Exploitation System (WFIA-ES). For this methodology, a crop pattern scenario is also set for a given year. Other uses of water are added to this scenario, such as urban and industrial supply, as well as the priority of water allocation. This scenario is simulated over a period of consecutive years, providing the value of the WF for the climate of different years.

Unlike the previous methodology (WFIA-FS), in the WFIA-ES the blue WF depends on the irrigation programme established for each crop. Specifically, irrigated areas have legal water allocations, which vary monthly, enabling access to a certain amount of water that depends on the crop mosaic within them. So, WF_Green_ plus WF_Blue_ usually is less than ET_c_ since it depends on the water allocations. If sufficient water resources are available, taking into account the concurrent water uses and the existing hydraulic infrastructure, then the supplies for irrigation are complete and coincide with the water allocations. Otherwise, there are water deficits in some irrigated areas and the difference between WF_Green_ plus WF_Blue_ and ET_c_ increases.

**Fig 2:**
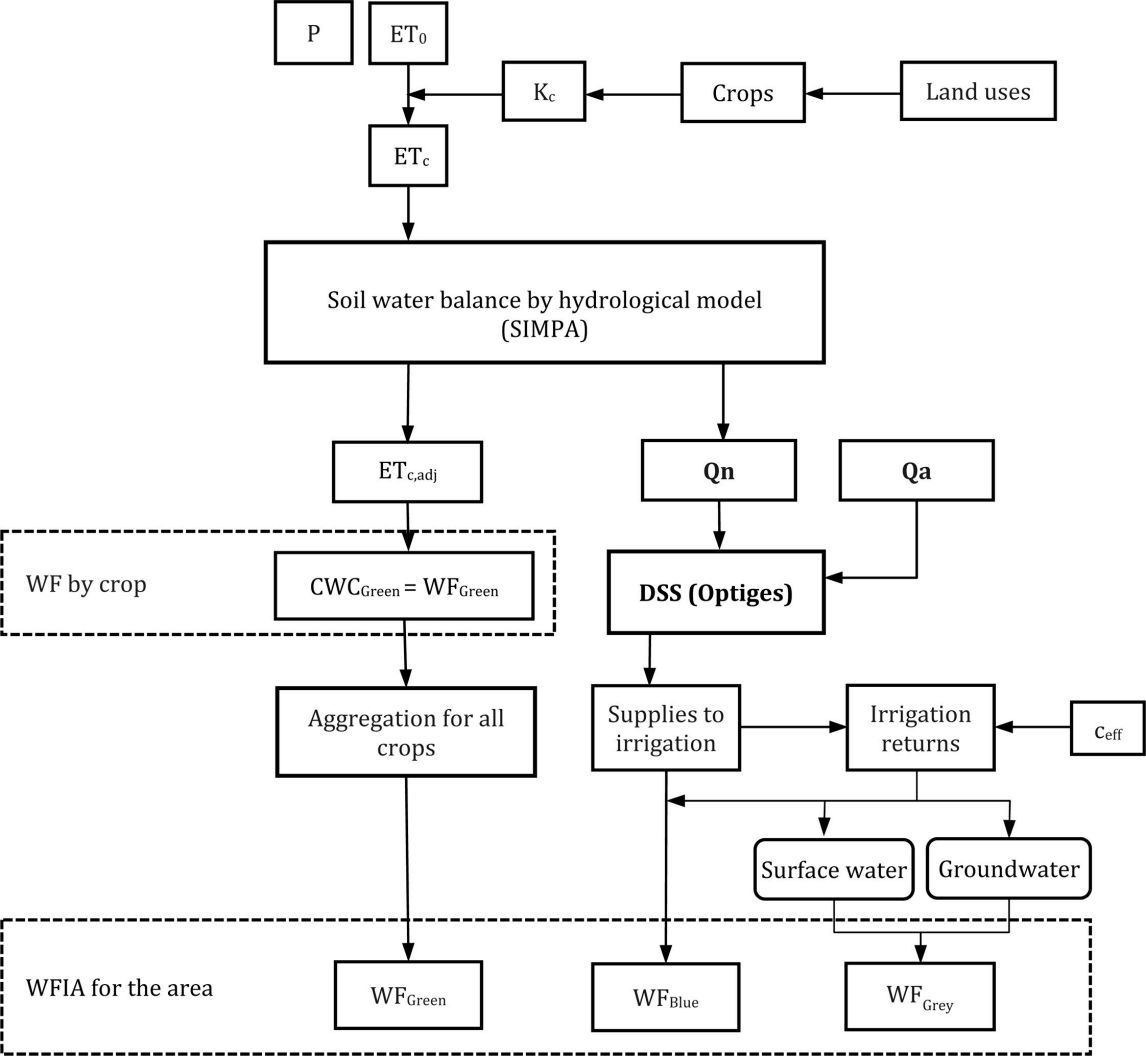
Methodological scheme for WFIA-ES accounting.

This methodology begins with the SIMPA distributed hydrological model. It provides temporal series of ET_c,adj_, run-off and recharge of the aquifers. The WF_Green_ is calculated using the ET_c_ and ET_c,adj_ series of the crops (Eq 2), as in the WFIA-FS methodology. The run-off and recharge series of the aquifers are the blue water resources of the basin (natural water resources, Qn [volume/time]). These series of blue water resources are used in the following step: modelling of the integrated system of water resources. This kind of modelling is usually carried out with a Decision Support System (DSS), which simulates a water network with water resources inputs and water uses [39]. The DSSs represent the interrelations between the main elements of the water exploitation system: rivers, reservoirs, lakes, aquifers, intakes, uses, desalination plants, return flows from water uses, etc. [40]. The main results are the series of the supply to irrigation [volume/time] and their return flows [volume/time], which are used in this methodology to account the WF_Blue_ and WF_Grey_, respectively. The water uses are located throughout the water network, as potential demands (water allocations). In other words, the maximum optimal values are defined and they are reached completely only when the resource is available in the area in which they are located. The natural water resources are incorporated into the water network at the locations where they are generated; for instance, inputs to reservoirs [41]. As stated previously, DSS models also allow the incorporation of non-conventional water sources (Qa [volume/time])—such as desalination and/or transfers—into the exploitation systems (Fig 2). Finally, the DSSs incorporate the return flows from the water uses into the same water network; therefore, when the topological conditions are appropriate, these water volumes can be reused in another water use (e.g. an irrigation area located downstream), preventing double-accounting in the WF_Blue_ calculation [18].

The DSS used in this work is the so-called Optiges (Fig 2), the optimisation module of AquatoolDMA, a platform to simulate water exploitation systems that is widely used around the world due to its versatility [39, 42, 43, 44, 45]. This DSS has been tested sufficiently in the basin under study [46, 47], since it is able to establish in the exploitation system the order of priority among the different concurrent water uses according to the Spanish water planning law [48, 49]. This order of priority is as follows, from greater to lesser priority: environmental requirements, urban-tourism, irrigation and industrial demands. This scheme of priorities at the operational level allows the DSS to provide resources to only one use when high-priority uses are already covered with a guaranteed level specified by law for each one of them [49]. So, this DSS distributes water resources among different concurrent water uses on a monthly basis, taking into account the topology of the water network and following three criteria: i) environmental requirements (flows and consumption by wetlands) as established by the WFD [50]; ii) the supply of available water resources, following the established order of priority of uses so as to distribute deficits between demands in times of scarcity [51]; and iii) the storage of maximum water volumes in reservoirs once the two former criteria are met.

The WF_Blue_ is determined as the series of water supplied less the series of return flows not consumed by crops, whereas the WF_Grey_ needs to be calculated for each series of irrigation return flows, using the following expression (Eq 6):

$${\text{WF}}_{\text{Grey}}=\text{max}\left[\text{k}\right]\left[\frac{{\text{Q}}_{\text{eff}}\cdot {\text{c}}_{\text{eff}}\left[\text{k}\right]]}{\left({\text{c}}_{\text{max}}\left[\text{k}\right]-{\text{c}}_{\text{nat}}\left[\text{k}\right]\right)}\right]\left[\text{volume}/\text{time}\right]$$ (6)

The pollutant load is determined from a return flow (Q_eff_ [volume/time]) and the concentration (c_eff_ [mass/volume]) of the different pollutant *k* substances that it contains. The Optiges DSS provides return flows (Q_eff_) and specifies the water body into which they are incorporated, being able to distinguish between surface and groundwater. The concentration of each pollutant comes from measurements of crop leachates (c_eff_). The maximum permissible concentrations for each pollutant (c_max_) are established by law for the studied river basin, whereas the natural concentrations (c_nat_) are defined by measurements of un-altered water bodies in the studied river basin [52]. Finally, the WF_Grey_ value for each irrigation return flow is also determined by the critical pollutant [6, 35].

### Case study

**Fig 3:**
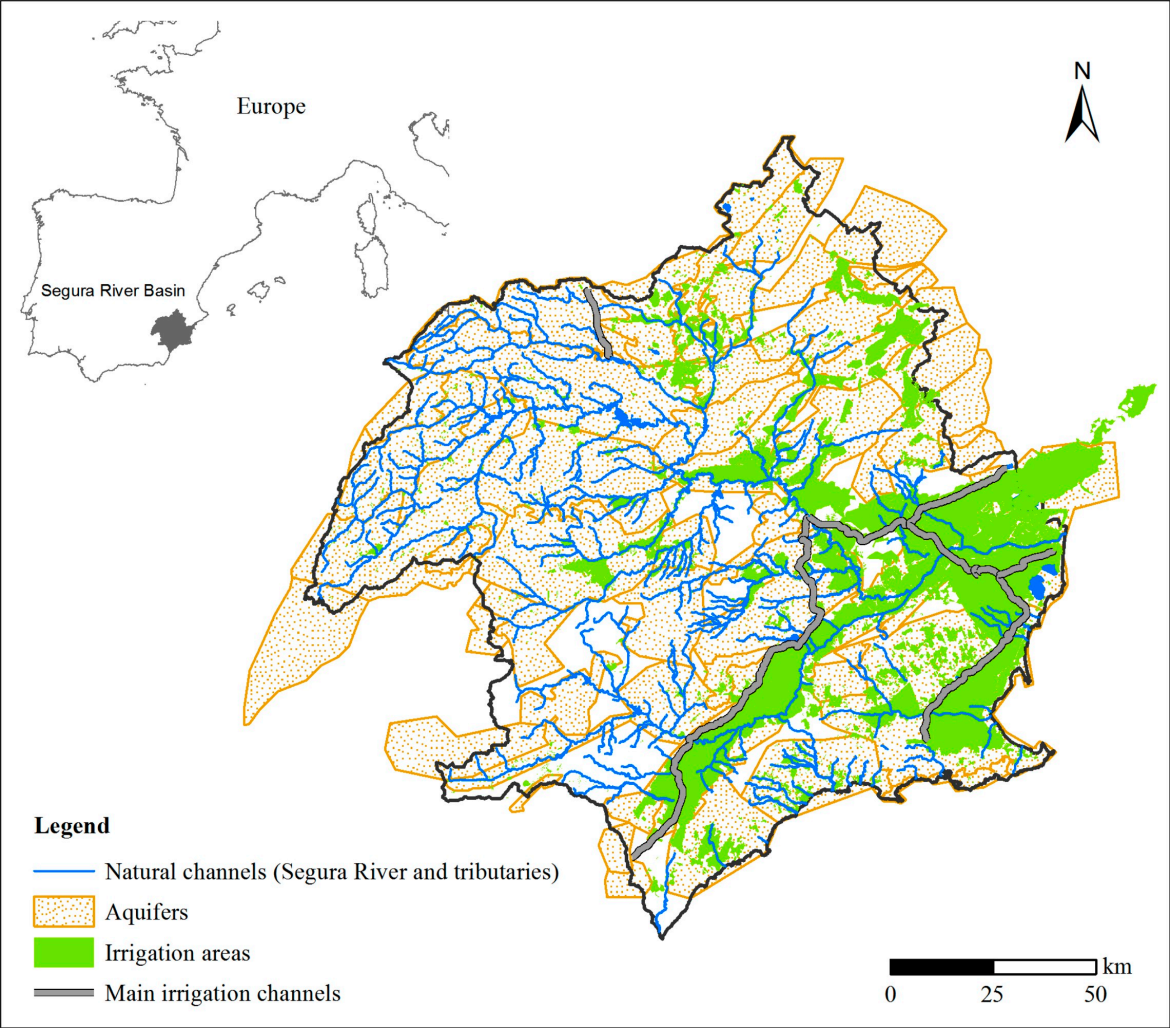
Location of the Segura River Basin, and its main characteristics in relation to the irrigation sector.

The Segura River Basin (SRB) district, which includes the SRB and other small coastal catchments without permanent streamflows, is located in South-eastern Spain and covers an area of 18 740 km^2^ (Fig 3). The co-existence of good-quality soils, a semi-arid climate and water resources, both surface and groundwater, has fostered the development of one of the most productive irrigated-agriculture systems in Europe [53]. Additionally, the horticultural sector is extremely advanced, with major exports, and there is also an important associated agro-industrial cluster. Currently, irrigated crops occupy about 262 393 ha, divided into 75 irrigation districts, called "Agrarian Demand Units". Irrigation uses over 85% of the available water resources [54]; regarding the method of irrigation, 73% is by drip irrigation, 25% by gravity and 2% by sprinkling. The origin of the water for irrigation is variable according to the hydrological year conditions, being, on average, 29% rainfall-runoff (superficial water), 20% inter-basin water transfer, 38% groundwater, 7% treated wastewater and 6% desalinated seawater. The second-greatest use by volume is the urban sector (14%), which supplies both the permanent population of around two million people and the strong tourism sector on the coast, especially in summer. Industry represents barely 1% of the water demands, although a large part of the industrial activity is directly connected to the urban distribution network [26].

**Fig 4:**
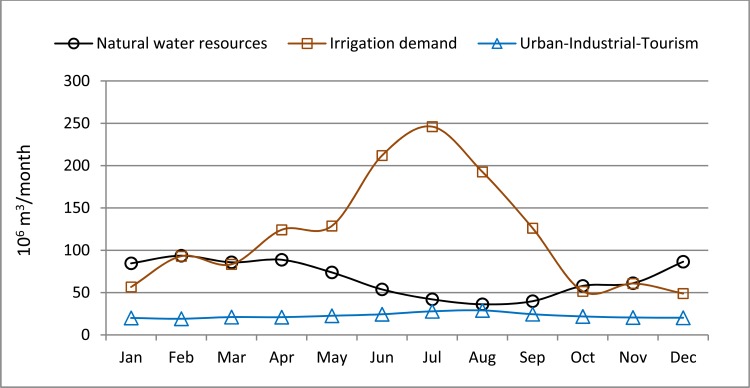
Average intra-annual variability of natural water resources for the period 1940–2010, and the water uses for the year 2015: irrigation demand, urban, industrial and tourism.

The average precipitation is 400 mm/year [55], although with a strong variability in space and time: it can be over 1000 mm/year in the North-western areas and under 200 mm/year on the coast. Maximum precipitation occurs in the winter and spring months (December-May), whereas precipitation is rare in the summer (June-September). The climate conditions lead to a noticeable seasonal pattern in the natural water resources available in the SRB, which is the opposite of that of the water demands of agriculture and tourism (Fig 4). This intra-annual gap between resources and demands, together with the frequent droughts, has led to the construction of important hydraulic infrastructures, such as channels and reservoirs, to connect irrigated lands. The capacity of the reservoirs (over 1100 10^6^ m^3^) is larger than the mean annual surface water resources. This is very important with regard to accumulation of water for drought periods and in case of increasing demands, and to minimise the damage resulting from floods. However, the supply problems have not yet been solved because there is no continuous excess of water resources available to be stored. An important transfer system from the Tagus river basin started to operate in 1979, to provide the SRB with additional resources [56]. Also, seawater desalination has been implemented [57], together with wastewater treatment and its direct reuse in irrigation [58, 59]. Despite the measures put in place to increase the available resources and the numerous management plans aimed at saving resources, such as the modernisation of irrigation systems, the SRB is currently the only river basin with a structural deficit in Spain, as acknowledged by authorities and institutions [26, 47]. This implies occasional supply deficits that are only diminished by the over-exploitation of aquifers [60].

### Data

The scenario for both methodologies was the year 2015. The crop pattern used to determine the crop evapotranspiration under standard conditions (ET_c_) for both methodologies was the Corine Land Cover 2000 classification of soil uses [14, 61]. This distribution of crop irrigation is representative of that year (2015) since the irrigated area and the crop pattern have not changed substantially since 2000. In fact, due to the scarce resources available, it is not possible to increase the irrigated area, only to maintain it [26]. The distribution of the irrigated areas, grouped according to the productive orientations, is: citrus (29%), vegetables (28%), stone-fruit trees (16%), vineyards (8%), olive trees (7%), almond (5%), intensive horticultural crops (2%), winter cereals (2%) and others (3%) [26]. The climatic data used in both methodologies cover the period 1940–2010.

The SIMPA hydrological model considers this crop scenario and the range of climatic data specified, providing the WF_Green_ series for both methodologies. However, regarding the blue water, WFIA-FS determines the WF_Blue_ series as the water deficit in the irrigated areas, whereas WFIA-ES uses the modelling of the integrated system of water resources. The year 2015 is the reference year for the design of the water exploitation system in the WFIA-ES methodology [50]. The irrigation water demand is defined according to the crop groups in the Basin Plan [26], which is based on the calculations of the National Irrigation Plan [62]–this considers the theoretical gross demand (the result of the net needs of each crop and the irrigation efficiency) and the allocation index of the agrarian demand unit. The crops of the basin are slightly under-endowed, their average supply being 75–90% of the theoretical gross demand. The modelling of the water exploitation system also considers environmental requirements, and urban and industrial uses. The environmental requirements are the ecological flows and the water volumes destined to the conservation of wetlands [63, 64]. Urban uses include the urban supply and tourism, and industrial uses are those that are unconnected to the urban network (Table 1). Irrigation constitutes by far the largest volume in the case study (more than 80% of the water used). The available water resources include the natural ones (the mean of the results of the SIMPA model), the resources from transfers, those from seawater desalination and the return flows that likely can be reused [65].

**Table 1:** Average data (10^6^ m^3^/year) used in the modelling of the water exploitation system. The environmental requirements, water demands and desalination capacity are for the year 2015; the transfer is the average volume transferred during the period 1979–2015; the surface and groundwater resources were provided by the SIMPA model for the period 1940–2010.

Envionmental requirements	Environmental flows^a^	0
	Consumption by wetlands	32
Water demands	Water supplies to urban and tourism demands	253
	Industrial	21
	Irrigation	1541
Water resources	Surface and groundwater resources (natural)	1010
	Desalination^b^	334
	Transfer	354
	Reuse^c^	80–120

^a^ Environmental flows are established in most of the river course except for the river mouth, where a null environmental flow is set, hence representing no demand. This does not mean that it is always zero in the river mouth but it has no priority of use.

^b^ Maximum desalination capacity of the basin. The desalination volume in the modelling depends on the natural water resources availability. In years of low availability of natural water resources, the volume of desalination water is highest, whereas in wet years the volume decreases since the natural water resources are used instead of desalinated water, which is more expensive.

^c^ The volumes that could be reused come from the returns of the demands. Therefore, they are not constant over time and their maximum value occurs when the demands are fully supplied.

The pollutants considered in both methodologies are nitrate and phosphate. For the WFIA-FS, the values of the application of fertilisers (AR) were established according to the practical guide on crop fertilisation in Spain [66], which establishes the mean values for each type of crop, taking into account the monthly pattern of the fertiliser application. The digital elevation map used to produce the slope map is available from the Spanish National Geographic Information Centre (http://www.ign.es/).

In the WFIA-ES methodology (Eq 6), the nitrate and phosphate loads employed were based on the study by [67], in which the pollutant concentrations in leachates of irrigated crops are discussed. These loads in the leachates showed high variability (as corroborated by other studies, such as those of [68, 69, 70]), underlining the fact that the larger the return flow in relative terms, the lower the concentration of nutrients (c_eff_) because they are more diluted. Therefore, this variability was introduced by a linear interpolation of pollutant concentrations for the range of percent returns in the basin: 5–15%. Thus, for the concentration of nitrate, the range of variability was established as: between 173 (mg/L), for return flows of 5%, and 28 (mg/L), for return flows of 15%. The same range of percent returns was used for the phosphate (see Table 2). These percentages were based on the results of [67] and on data published by the water board for previous investigation in different irrigation areas [26, 46, 47, 65].

**Table 2:** Fertiliser loads applied (AR: nitrate, phosphate), with the percentage (α) that reaches the natural water bodies (WFIA-FS), and the concentrations of pollutants considered (c_eff_: nitrate, phosphate) in the irrigation returns (WFIA-ES). Maximum acceptable pollutant concentrations (c_max_) specified in the Spanish law (WFIA-FS and WFIA-ES). Natural concentrations (c_nat_) are the measurements in the unaltered water bodies in the SRB.

	WFIA-FS		WFIA-ES	Maximum acceptable pollutant concentrations (c_max_) (Spanish law)		Natural concentrations (c_nat_) (measurements in the unaltered water bodies)	
Pollutant (*k*)	AR (kg/ha)	α	Concentrations (mg/L) of the pollutants (*c*_*eff*_) in the irrigation returns interval (5%-15%)	Surface water bodies	Groundwater bodies	Surface water bodies	Groundwater bodies
Nitrate	10–260	3% - 10%	173–28	< 25 mg/L NO_3_^-^	< 50 mg/L	0 mg/L	0 mg/L
Phosphate	5–80	3% - 10%	1.180–0.008	< 0.4 mg/L PO_4_^3-^	Not contemplated	0 mg/L	0 mg/L

Regarding c_nat_, the measurements made in the unaltered surface and groundwater bodies showed that the nitrate and phosphate concentrations were around zero [71], which allowed the establishment of null values for the natural concentrations of both pollutants.

For aquifers, only nitrate was included in the calculation of the WF_Grey_, since the current law does not contemplate a limitation for phosphates in aquifers, whereas both pollutants were analysed in surface water bodies (Table 2). In the latter case, the pollutant that needs a greater volume of water in order to be assimilated is the one that establishes the value of the grey water footprint (called the critical pollutant), since this volume is capable of diluting both pollutants [35, 37].

## Results

The water footprint of irrigated agriculture (WFIA) results for the SRB are presented below, according to the methodology (WFIA-FS and WFIA-ES). In addition, a comparison of the two methodologies is included in the final section.

### Water footprint of irrigated agriculture with full supply (WFIA-FS)

The WFIA-FS had a mean value of 4403 10^6^ m^3^/year, ranging between 3770 and 4563 10^6^ m^3^/year. The breakdown of the WFIA-FS revealed that the blue component had the greatest weight (67%), more than double that of the green (28%), while the grey component accounted for barely 5% of the WFIA-FS (Table 3). These percentages are very similar to those obtained for the irrigated lands of the Guadiana river basin (close to the SRB, with a similar climate), to which the same methodology was applied, using the CROPWAT model for the soil water balance [14].

**Table 3:** Main statistics of WFIA-FS, WFIA-ES and their respective components, and the relative values of WFIA-FS (%) with respect to WFIA-ES.

**WFIA-FS (10**^**6**^ **m**^**3**^**/year)**	**Main statistic**	**WF**_**Green**_	**WF**_**Blue**_	**WF**_**Grey**_	**WFIA-FS**
	Average	1214	2957.1	231.4	4402.5
	Maximum	2036.6	3631.7	364.4	4563.4
	Minimum	744.2	1929.8	29	3769.8
	Standard deviation (Sd)	301.6	377.9	74.5	125.1
**WFIA-ES** **(10**^**6**^ **m**^**3**^**/year)**	**Main statistic**	**WF**_**Green**_	**WF**_**Blue**_	**WF**_**Grey**_	**WFIA-ES**
	Average	1214	1404.8	254.9	2873.7
	Maximum	2036.6	1422.1	245.8	3717
	Minimum	744.2	1213.6	258.3	2216.6
	Standard deviation (Sd)	301.6	30.4	1.88	315
**Relative values of the WFIA-FS (%)**		**WF**_**Green**_	**WF**_**Blue**_	**WF**_**Grey**_	**WFIA**
	Average	-	210%	91%	153%
	Maximum	-	255%	148%	123%
	Minimum	-	159%	11%	170%

In the relative values of the WFIA-FS, WF_Green_ was not introduced into this analysis because it is the same in both approaches. Full data available at: WFIA-FS: https://doi.pangaea.de/10.1594/PANGAEA.892557; WFIA-ES: https://doi.pangaea.de/10.1594/PANGAEA.892558

**Fig 5:**
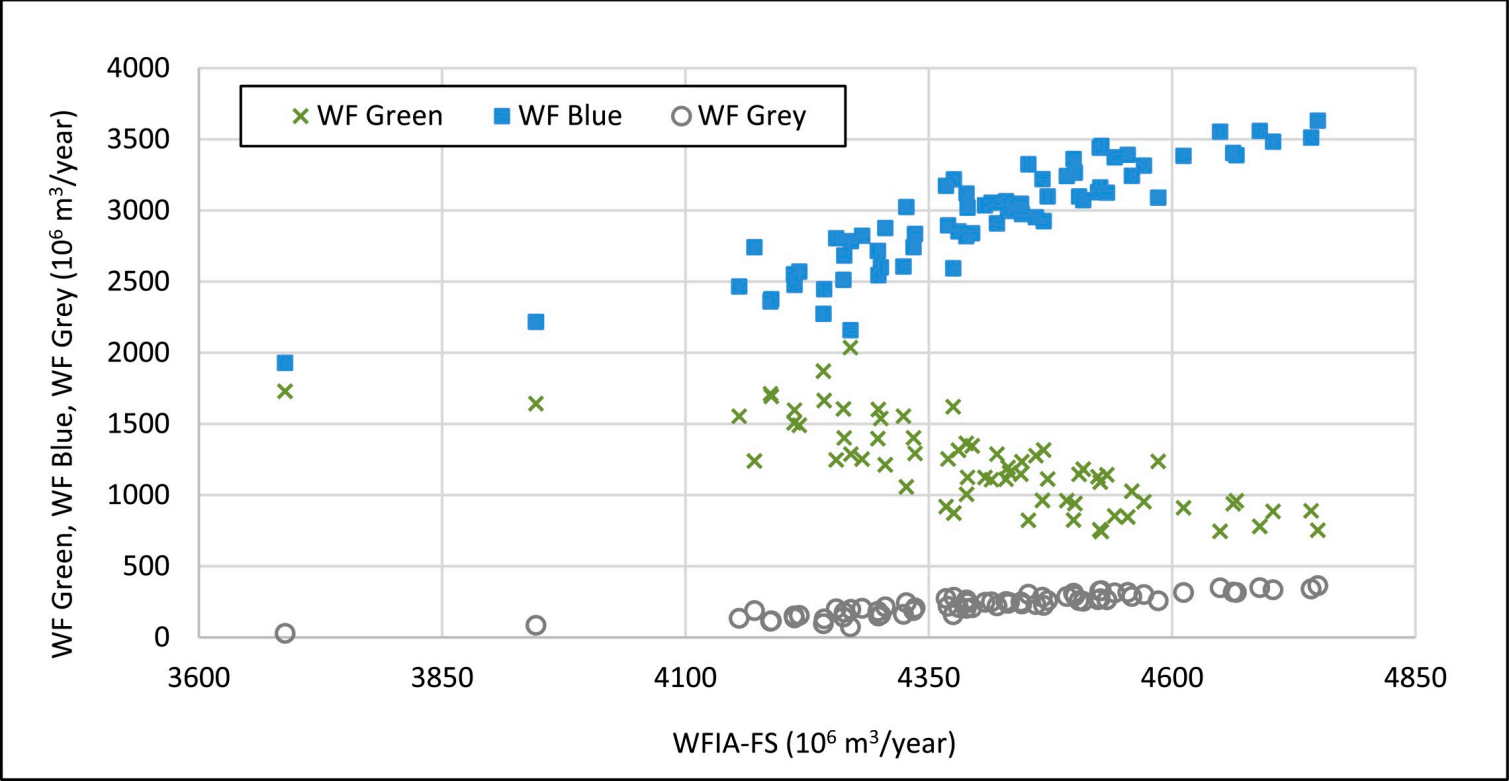
Relationship between the WFIA-FS and its components (10^6^ m^3^/year).

The standard deviation of the WFIA-FS was smaller than those of the green and blue components of the WFIA-FS (Table 3). In wet years there is more water available in the soil and the crop evapotranspiration under non-standard conditions (ET_c,adj_) is greater (greater WF_Green_). Therefore, water deficits are lower and WF_Blue_ decreases. This is a consequence of the methodology applied, since the sum of WF_Green_ and WF_Blue_ has to be ET_c_. So, this results in a negative correlation between these two components and the trade-off between them balances the aggregated value of WFIA-FS. WF_Grey_ had a greater relative variability, resulting from the amount of irrigation applied, since the slope and the AR do not vary with time. In years with less precipitation WF_Grey_ would increase, as the amount of irrigation water applied—that washes the fertilisers into the water bodies—would also increase (Fig 5). Finally, the WFIA-FS and its three components behaved like normal random variables (they derive from the same data series: evapotranspiration and precipitation), checked with the Shapiro-Wilk test and the Anderson-Darling test at 1% [72], and the results can be expressed in probabilistic terms or with confidence intervals. This information enables one to know the probability of obtaining a WFIA-FS higher than a given value; for example, there is a 95% probability of obtaining a WFIA greater than 4157 10^6^ m^3^/year.

### Water footprint of irrigated agriculture with exploitation system (WFIA-ES)

The WFIA-ES had a mean value of 2874 10^6^ m^3^/year, ranging from 2217 to 3717 10^6^ m^3^/year. The blue component’s weight (49%) was greater than that of the green (42%), whereas the grey component accounted for 9% of the WFIA-ES (Table 3). The standard deviation of the WFIA-ES depended mainly on the green component; the standard deviations of the other two components (blue and grey) were much lower. The low variability of the blue WF is the result of the agricultural water management in the basin studied, and is basically due to the legal water allocations of the irrigated areas and the exploitation system included in this methodology through the DSS model. This model considered the regulation of surface water by reservoirs, the functioning of desalination plants in drought periods and/or access to non-renewable groundwater in over-exploited aquifers of some irrigated lands. So, the WFIA-ES only had a positive, significant relationship with WF_Green_ and a non-significant one with the other two components. Finally, WF_Blue_ and WF_Grey_ had a positive, significant relationship, because when supply increases, so do the return flows.

The WFIA-ES and the WF_Green_ component behaved like normal variables, according to the Shapiro-Wilk and Anderson-Darling tests at 1% [69]. Since WF_Blue_ and WF_Grey_ did not fit a normal distribution, their fits to other distributions were tested: WF_Blue_ fitted a Cauchy distribution, whereas WF_Grey_ fitted a Gumbel-Min distribution best. Therefore, as with the previous methodology, the results can be presented in probabilistic terms and allow the performance of statistical inferences. But, unlike WFIA-FS, the blue and grey components had a distribution with a heavy left tail, representing the years with deficits. The statistical behaviour of the WF shows that WF_Green_ is a random variable that depends essentially on natural phenomena whereas WF_Blue_ and WF_Grey_ are random variables that are influenced more by anthropic actions.

## Discussion

Two methodologies were applied to the same case study. A comparison of the values obtained, in the form of index numbers, is presented in the last rows of Table 3. The first fact to be underlined is that the average value of the total WFIA-FS (4403 10^6^ m^3^/year) is 53% greater than for the methodology that incorporated the exploitation system (2874 10^6^ m^3^/year). The same comparison of the two methodologies shows an increased discrepancy, of 70%, for the minimum values, whereas the maximum values are closer, with a difference of only 23%.

The WF_Blue_ was mainly responsible for these differences, as the methodology with full supply gave values for this component that clearly doubled (210%) those of the new methodology proposed. Representation of the WF_Blue_ series for both methodologies and their comparison with the series of available natural water resources in the SRB over the same period shows that there would not be sufficient water to cover the WF_Blue_ obtained when taking into account full supply, not even when considering over-exploitation of aquifers and/or alternative sources. It is clear then that the WF_Blue_ values, and hence the WFIA, are overestimated with WFIA-FS, and the blue water consumption values provided are impossible in the SRB owing to the actual availability [17, 19]. This comparison demonstrates that the green and blue components of the WFIA have to be accounted by following the hydrological principles [17], as the WFIA-ES does provide WF_Blue_ values that are compatible with the availability of resources in the SRB.

**Fig 6:**
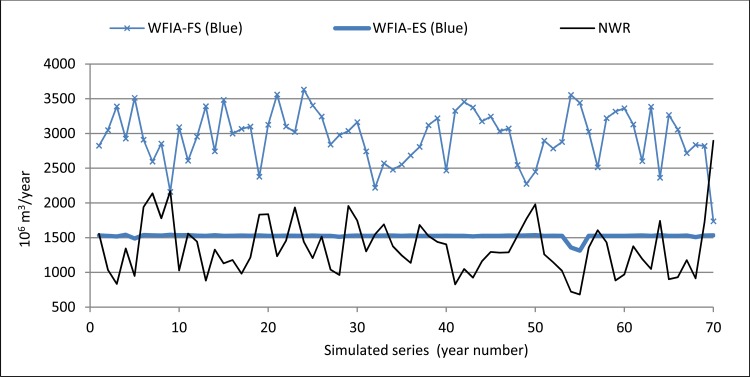
WFIA_Blue_ values with both methodologies and natural water resources (NWR).

**Fig 6**also shows the high variability of the blue component of the WFIA-FS and its negative relationship with the series of available natural water resources, which is hard to explain in physical terms. This contrasts with the relative stability of the WF_Blue_ calculated using WFIA-ES—which, as stated previously, is the result of water allocations, reservoir regulation, desalination and aquifer over-exploitation.

The average values of WF_Grey_ were alike for the two methodologies, being 9% higher in WFIA-ES, although the extreme values of WF_Grey_ differed between the two approaches. Both methodologies, in accordance with the findings in previous studies [16], yielded a positive relationship between the WF_Grey_ and WF_Blue_ values: linear for WFIA-FS and non-linear for WFIA-ES. Regarding the critical pollutant, nitrate was responsible for 80% of the WF_Grey_ in the WFIA-FS methodology, a value that increased to 97% when calculated by the WFIA-ES methodology. These differences can be negligible if the considerations adopted are taken into account: the fertiliser load applied does not depend on the year (WFIA-FS) and the concentrations and their variability in the returns are introduced in a simplified way (WFIA-ES). It should also be noted for the two methodologies used that not all the variability of the grey component is reflected in the final results, leaving out two important sources of uncertainty: the uncertainty of the data used and the maximum concentration (c_max_) that is set, which in this case depends on legal criteria. The choice of the c_max_ is crucial since it can modify the WF_Grey_ value substantially or even change the critical pollutant. In this sense, although the grey component serves to show the degree of contamination of the basin, it will always be fixed by the restrictive or lax limitations of the stipulated maximum concentrations.

Focusing the analysis on WFIA-ES, the proposed methodology addresses the appraisal of the WFIA at the level of the river basin from the hydrometeorological perspective, and considering the hydraulic characteristics of its water exploitation system. Therefore, it focuses on one of the components of scarcity, namely the water supplies. However, the social and productive structure of the river basin must not be forgotten, since it is the other component of the scarcity concept. Thus, droughts due to anomalous deficits in the supplies to water uses, known as operational droughts, are not produced exclusively by a decline in natural water resources (hydrological droughts), they are also produced by an excess of water demand or by inadequate water resources management, so that they are usually known as socio-economic droughts [73]. These considerations are relevant in river basins located in areas with low availability of water resources and concurrent water uses. They are especially pertinent when considering the likely increment in the frequency of socio-economic droughts in semi-arid areas caused by future population growth and climate change effects on natural water resources [74]. In this regard, the appraisal of the WFIA could be a useful tool in hydrological planning in the medium and long term, since irrigated agriculture will be the water use that suffers most directly the effects of the socio-economic droughts [22].

Finally, to address one of the limitations of this work, an improvement could be achieved by considering in more detail specific aspects of each crop in the irrigated areas, such as different irrigation strategies, the phenological stages of the crops and their yields. For this purpose, the water supplied would be disaggregated for the crop areas, obtaining a homogeneous indicator that would quantify the pressure on water resources exerted by each crop, regarding both quantity and quality. This information would be very useful with regard to knowing how the water availability affects the different crop yields, and could be used to support the design of land use policies.

## Conclusions

This study evaluated the WF of irrigated agriculture in a geographical area (the Segura River Basin, South-eastern Spain), proposing a new approach for its calculation (the water footprint of irrigated agriculture with exploitation system: WFIA-ES) that addresses key aspects criticised in previous works [17–19]. This approach considers the actual availability of water resources, the exploitation system that distributes them and the legal criteria for water management in irrigation. Moreover, as the amount of water (blue and green) incorporated into the modelling comes from the same simulation, and coincides with the precipitation that actually falls in the basin, it can be said that the methodology complies with the basic principles of hydrology (satisfying the law of continuity of mass).

The WFIA-ES methodology has been applied here together with the methodology that have been used generally in other works and that supposes a complete supply to match the irrigation demands (the water footprint of irrigated agriculture with full supply: WFIA-FS). Both methodologies used the same series of climatic data, the same land uses and the same soil balance model. So, the series of WF_Green_ obtained are the same. The two approaches gave similar average values of WF_Grey_, despite the differences in the calculation processes. In addition, for both methodologies, the values obtained for the grey component are directly proportional to the volume that returns and to the contaminant load, as established in the proposed formulation. The main differences are in the blue component. For the methodology that considers full supply (WFIA-FS) the WF_Blue_ value almost doubles that obtained using the methodology that recreates the anthropised water cycle (WFIA-ES). In fact, the value of WF_Blue_ for WFIA-FS exceeds the sum of the water resources available in the basin, and it is also decoupled from the hydrology of the basin. Moreover, there is an inversely proportional relationship between resource availability and WF_Blue_, which is difficult to explain in physical terms. So, this value could never be obtained in practice for a semi-arid basin. However, the blue water consumption values for WFIA-ES are in line with the actual availability of water. These results reveal that the values obtained using WFIA-FS are highly over-estimated. This methodology simply provides a maximum reference value that the WFIA could achieve if spatial and temporal availability were not a limiting factor.
